# Radiographic phenotyping of healthy and diseased aortas

**DOI:** 10.1186/s44348-026-00086-5

**Published:** 2026-10-13

**Authors:** Joel C. Boudreaux, Suleman Chaudhry, Nikahat Yasmine, Saubhagya Dhakal, Honghai Zhang, Milan Sonka, Andreas Wahle, Sarah Vigmostad, Abhay B. Ramachandra, William H. Thiel, Loren E. Smith, Jennifer Streeter, Rodrigo M. Bello, Arun K. Singhal

**Affiliations:** 1 Department of Cardiothoracic Surgery, University of Iowa, Iowa City, IA USA; 2 Department of Radiology, University of Iowa, Iowa City, IA USA; 3 Department of Electrical and Computer Engineering, University of Iowa, Iowa City, IA USA; 4 The Iowa Institute of Biomedical Imaging, University of Iowa, Iowa City, IA USA; 5 Roy J. Carver Department of Biomedical Engineering, University of Iowa, Iowa City, IA USA; 6 Department of Mechanical Engineering, Iowa State University, Ames, IA USA; 7 Department of Internal Medicine and The François M. Abboud Cardiovascular Research Center, University of Iowa, Iowa City, IA USA; 8 Department of Anesthesia, University of Iowa, Iowa City, IA USA

**Keywords:** Aortic dissection, Thoracic aortic aneurysm, Computed tomography angiography, Thoracic aorta (pathology), Phenotype

## Abstract

**Background:**

Ascending aortic diameter guides intervention for thoracic aneurysms. Many patients with acute type A aortic dissection (ATAAD) present with ascending aortic diameters below 5.5 cm, rendering diameter an insufficient risk discriminator. This study evaluated computed tomography angiography (CTA) morphology to identify predissection radiographic features associated with ATAAD.

**Methods:**

Control (*n* = 50, CTA for non-aortic indications), aneurysm (*n* = 61, thoracic ascending aorta [TAA] ≥ 4.5 cm), and predissection (*n* = 13, CTA within 3 years before dissection) groups were studied. Scans were interpreted by thoracic radiologists using predefined measurements.

**Results:**

Age and sex distribution differed overall among groups (*P* < 0.001 and *P* = 0.010, respectively). The aneurysm group was older than controls, but the age difference from the predissection group was not significant (*P* = 0.084). Maximum TAA diameter (MaxD) was 3.11 ± 0.30, 4.96 ± 0.52, and 3.89 ± 0.60 cm in control, aneurysm, and predissection groups respectively; all predissection MaxD values were below 5.5 cm (maximum, 4.9 cm). Ascending aortic length (AAL) was elevated in both disease groups (aneurysm group, 9.03 ± 1.08 cm; predissection group, 10.73 ± 1.41 cm; *P* < 0.001 vs. control group, 6.38 ± 0.75 cm). The AAL/MaxD ratio differed significantly between every pair of groups (control group, 2.06 ± 0.22; aneurysm group, 1.83 ± 0.21; predissection group, 2.80 ± 0.50; all pairwise P < 0.001), reflecting disproportionate elongation in predissection aortas. Aneurysm patients demonstrated diffuse thoracic dilation, whereas predissection group patients showed predominantly ascending aortic involvement.

**Conclusions:**

In a small exploratory study, predissection aortas displayed a distinct morphological phenotype characterized by disproportionate elongation, moderate dilation, and a relatively nondilated descending aorta—distinct from the diffuse dilation of aneurysm. Ascending aortic length and the AAL/MaxD ratio may provide complementary morphological information beyond maximal diameter alone.

**Supplementary Information:**

The online version contains supplementary material available at https://doi.org/10.1186/s44348-026-00086-5.

## Background

Acute type A aortic dissection (ATAAD) is a devastating and lethal disease with a mortality rate of over 50% [1–3]. Despite surgical advances, morbidity and mortality remain high. ATAAD is a partial-thickness tear involving the inner two layers of the aorta (intima and media) located in the thoracic ascending aorta (TAA). Surgical replacement of TAA aneurysms, the presumptive prodrome to ATAAD, carries substantially lower mortality risk when treated electively (3.5%) [4]. Elective surgical intervention for these aneurysms mitigates risk of ATAAD by prophylactic replacement. However, our ability to discern a priori at-risk patients is poor. Presently, guidelines define at-risk patients primarily by a maximal TAA diameter ≥ 5.5 cm [5]. Multiple lines of evidence demonstrate the inadequacy of using the diameter as a surrogate for dissection risk [6–10]: (1) most ATAAD occur at diameters below the 5.5 cm threshold (59% of type A dissections in the International Registry of Acute Aortic Dissection, IRAD, were below 5.5 cm when measured at dissection [7], and 96% of 25 patients with a CT before dissection were below 5.5 cm [8]); (2) emerging recognition of other TAA dimensions; and (3) observed asymmetry in both cystic medial degeneration and location of intimal tears relative to the site of maximal radius. Although elective replacement of the TAA is low risk, we do not know how to identify at-risk patients.

Increasingly, aortic length is developing as a complementary predictor of ATAAD risk as well as volume, sinotubular diameter, and tortuosity [7–9, 11]. In this study, we evaluated thoracic aortic morphology holistically by comparing three populations: nonaneurysmal controls (control group), patients with known TAA aneurysm (aneurysm group), and patients who developed ATAAD with available predissection computed tomography (CT) imaging (predissection group), aiming to identify consistent radiographic characteristics that distinguish these groups.

## Methods

### Ethics statement

This study was approved by the University of Iowa Institutional Review Board (No. 20250325 University of Iowa). Individual patient consent was waived due to the use of deidentified data and the retrospective nature of the study.

### Study cohort

A single-center retrospective review with all patients presenting for repair of ATAAD between 2019 and 2024 was performed. Between January 2019 and December 2024, a total of 151 patients underwent ATAAD repair at our institution. Thirteen patients (8.6%) with a suitable CT scan of the chest within 3 years prior to dissection were identified (predissection group) with a median of 165 days (range, 29–868 days). The clinical indications for predissection CT imaging were not available for review. Ethical approval for this study permitted access to radiographic images and basic demographics included in the radiographic interpretation (age, sex, height, and weight). The predissection group was restricted to those patients for whom diagnostic quality imaging could be retrieved, the majority being from outside institutions given the rural nature of our service area. These CT scans were collected, and aortic dimensions were reviewed by radiology. A representative control group was identified from randomly selected patients undergoing chest CT angiography (normal-sized aortas without aortic disease) during the same period. Patients with previous cardiac surgery and bicuspid valves were excluded. A third group of patients with known TAA aneurysms (defined in this study as maximal diameter ≥ 4.5 cm) undergoing surveillance CT scans was identified (aneurysm group). A total of 124 patients were included (predissection group, *n* = 13; control group, *n* = 50; and aneurysm group, *n* = 61) (Table 1).

**Table 1:** Patient demographics by group

Demographic	Control (*n* = 50)	Aneurysm (*n* = 61)	Predissection (*n* = 13)	*P*-value			
				Overall	Control vs. Aneurysm	Control vs. Predissection	Aneurysm vs. Predissection
Age (yr)	57.82 ± 18.14 (17–90)	68.80 ± 10.86 (38–85)	59.15 ± 15.52 (24–79)	< 0.001	< 0.001	0.954	0.084
Male sex	21 (42.0)	43 (70.5)	8 (61.5)	0.010	0.0047	0.344 (Fisher 0.230)	0.762 (Fisher 0.526)
Height (cm)^a^	170.84 ± 10.10 (152.4–193)	174.39 ± 9.31 (157.5–188)	168.62 ± 8.32 (157–183)	0.054	NS	NS	NS

Values are presented as mean ± standard deviation (range) or number (%)

^a^Height was available for 60 of 61 aneurysm patients

### Image acquisition and analysis

Helical CT angiography (CTA) was performed with dynamic iodinated contrast infusion and bolus tracking in the ascending aorta. Most predissection scans in the predissection group were performed at referring facilities; only studies with adequate diagnostic quality—free of significant motion artifact and with sufficient intravascular opacification—were included. Predissection CT scans, the majority of which were obtained at outside referring facilities, had ECG gating status that could not be centrally confirmed. All CT scans performed at the University of Iowa were ECG gated. Images were reconstructed at 1.0 to 3.0 mm slice thickness using a standard vascular kernel and exported to a dedicated postprocessing workstation.

### Radiographic interpretation

All thoracic CTAs were retrospectively evaluated on a dedicated workstation (Vitrea Advanced Visualization, Canon Medical Systems) using multiplanar reconstructions (MPR) and 3D imaging. Each study was reviewed by two radiology fellows and a board-certified thoracic radiologist with 23 years of experience. Scans were assessed for aortic abnormalities; patients with any acute aortic abnormality, prior cardiac surgery, or bicuspid aortic valve were excluded from all three groups: Control, Aneurysm and Pre-dissection. In the Pre-dissection group, no acute aortic abnormality refers to the pre-event study and not the study at the time of dissection.

### Diameter and length measurements

**Fig. 1:**
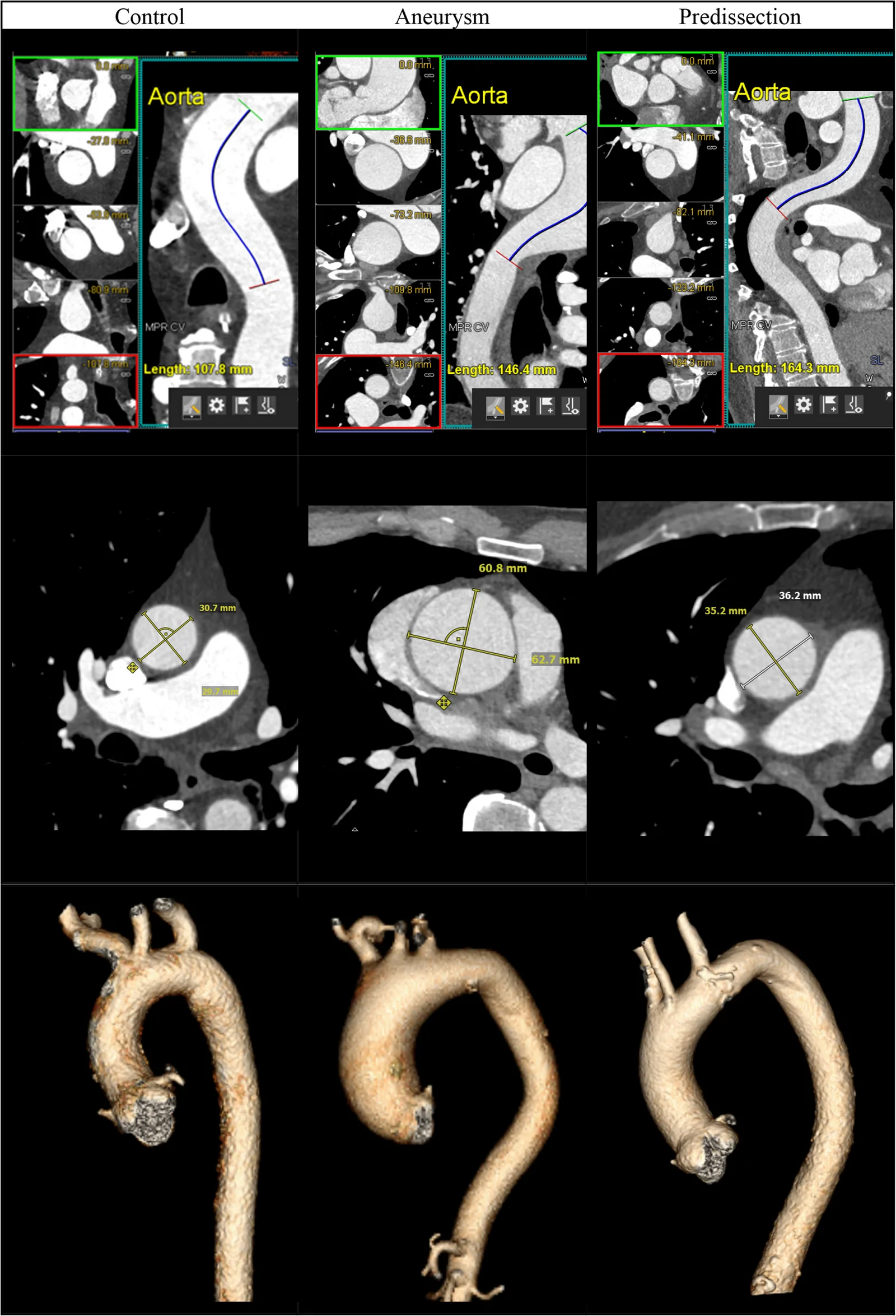
Representative radiographs of a control (left), aneurysm (middle), and predissection (right) aorta. Length was measured along a centerline through the midpoints of the ascending aorta. The value shown as “Length” in each panel is the ascending aortic length, the distance from the sinotubular junction (STJ) to the base of the innominate artery (control, 68.8 mm; aneurysm, 101.6 mm; predissection, 112.4 mm). Numbers along the left margin of each panel give the position of each cross-section along the centerline, in mm. Diameters were measured on cross-sections perpendicular to the centerline. The illustrated predissection CT was obtained at an outside referring facility; ECG gating status could not be confirmed

DICOM datasets were imported and vessel segmentation was performed using automated intensity thresholding with manual correction as needed. A semiautomated tool generated a 3D centerline from the aortic root to the end of the chest CT scan or, when an abdominal CT was also available, to the iliac bifurcation. Measurements extended from the aortic root to the level of the diaphragm. Anatomical landmarks were confirmed manually, and curved MPR reconstructions orthogonal to the centerline ensured accurate cross-sectional measurements (Fig. 1). Aortic diameters were measured on double-oblique cross-sectional planes perpendicular to the vessel centerline (outer-to-outer wall) at predefined levels: coronary cusps, sinotubular junction (STJ), mid-aortic arch (zone 1), mid-descending thoracic aorta, and diaphragm. The maximum ascending diameter (MaxD) was the largest diameter measured in the ascending aorta from the STJ to the origin of the innominate artery, with the STJ included in that region. Ascending aortic length (AAL) was the centerline distance from the STJ to the origin of the right brachiocephalic artery. All measurements were recorded in centimeters. Interobserver reproducibility was assessed in a randomly selected subset of 98 patients, each measured independently by two observers with the second observer blinded to the first measurements. Complete paired readings, confirmed by matching accession number, were available for 72 patients (25 control, 40 aneurysm, and 7 predissection), and all seven parameters were analyzed in these 72 pairs (Table S1). Reproducibility was quantified using the intraclass correlation coefficient (ICC; ICC(A,1), two-way absolute agreement, single measures) and Bland–Altman analysis (mean bias ± limits of agreement). Coefficient of variation was calculated as the standard deviation of paired differences divided by √2 times the grand mean, enabling comparison of reproducibility across measurements of different scale (Table S1).

### Statistical analysis

Demographic (age, height, and sex) and morphologic variables are reported as mean ± standard deviation with full range (minimum to maximum). Categorical variables are reported as counts with percentages. Group comparisons for continuous variables were performed using one-way analysis of variance (ANOVA). Post hoc pairwise comparisons were conducted using the Tukey honest significant difference test. Overall differences in sex distribution across the three groups were assessed using the chi-square test; pairwise comparisons of sex distribution used the continuity-corrected (Yates’) chi-square test, with Fisher exact test results also reported given small cell counts in some comparisons. Comparisons between patients with and without predissection CT (Table S3) used the two-sample t-test (equal variance assumed) for age and height and the Fisher exact test for sex. Paired predissection and at-dissection measurements (Fig. 4) were compared using the paired t-test, and associations between the interscan interval and morphological change were assessed using Pearson correlation. A *P*-value of < 0.05 was considered statistically significant. Age, biological sex, and height are recognized confounders of aortic dimensions. To assess the robustness of primary findings, an analysis of covariance (ANCOVA) was additionally performed for AAL and the AAL/MaxD ratio, with age, sex, and height entered as covariates. Given the unequal sample sizes across groups, particularly the relatively small predissection cohort, ANCOVA results are reported narratively in the text rather than tabularly and were found to be consistent with the unadjusted ANOVA findings. Aortic morphology was assessed using three complementary approaches: (1) absolute measurements in centimeters (Table 2); (2) measurements normalized to the patient's MaxD, expressed as a dimensionless ratio (Table 3); and (3) measurements indexed to patient height (Table S2). Normalization to MaxD was selected as the primary hypothesis-driven analysis, as it permits direct comparison of the spatial relationships between aortic segments independent of overall aortic size. Receiver operating characteristic (ROC) curve analysis was performed to assess the discriminative performance of the ascending aortic length-to-maximal-diameter ratio (AAL/MaxD) between groups. Optimal cutoffs were selected by maximizing the Youden index (J = sensitivity + specificity − 1). Ninety-five percent confidence intervals for the area under the curve (AUC) were computed by 2,000-iteration stratified bootstrap resampling (percentile method, random seed 42), consistent with the Table S4 footnote. Analyses were performed in Python (version 3.14.0) using scikit-learn (version 1.9.1) for ROC curve and AUC estimation, NumPy (version 2.5.3) for the bootstrap resampling procedure, SciPy (version 1.18.1) for ANOVA and Tukey comparisons, and statsmodels (version 0.15.0) for the ANCOVA.

**Table 2:** Aortic morphology measurements by group

Variable	Control (*n* = 50)	Aneurysm (*n* = 61)	Predissection (*n* = 13)	*P*-value			
				Overall	Control vs. Aneurysm	Control vs. Predissection	Aneurysm vs. Predissection
Maximum STJ diameter (cm)	2.81 ± 0.31 (2.0–3.4)	3.94 ± 0.61 (2.7–6.6)	3.41 ± 0.55 (2.7–4.1)	< 0.001	< 0.001	< 0.001	0.0022
Maximum ascending diameter (cm)	3.11 ± 0.30 (2.3–3.5)	4.96 ± 0.52 (4.6–6.6)	3.89 ± 0.60 (3.1–4.9)	< 0.001	< 0.001	< 0.001	< 0.001
Aortic arch (cm)	2.68 ± 0.32 (1.7–3.2)	3.34 ± 0.42 (2.6–4.4)	2.82 ± 0.41 (2.1–3.7)	< 0.001	< 0.001	0.433	0.0001
Descending aortic diameter (cm)	2.30 ± 0.31 (1.5–3.0)	3.10 ± 0.76 (1.9–6.0)	2.43 ± 0.36 (1.9–3.0)	< 0.001	< 0.001	0.759	0.0007
Aortic diameter at diaphragm (cm)	2.13 ± 0.34 (1.2–3.5)	2.80 ± 0.66 (1.7–5.2)	2.23 ± 0.26 (1.8–2.6)	< 0.001	< 0.001	0.821	0.0013
Ascending aorta length (cm)	6.38 ± 0.75 (4.5–7.9)	9.03 ± 1.08 (7.0–11.9)	10.73 ± 1.41 (8.3–12.3)	< 0.001	< 0.001	< 0.001	< 0.001

Values are presented as mean ± standard deviation (range)

*STJ* sinotubular junction

**Table 3:** Aortic morphology normalized to MaxD

Variable	Control (*n* = 50)	Aneurysm (*n* = 61)	Predissection (*n* = 13)	*P*-value			
				Overall	Control vs. Aneurysm	Control vs. Predissection	Aneurysm vs. Predissection
STJ/MaxD	0.90 ± 0.06 (0.8–1.0)	0.80 ± 0.10 (0.6–1.0)	0.88 ± 0.07 (0.8–1.0)	< 0.001	< 0.001	0.537	0.0055
Arch/MaxD	0.86 ± 0.07 (0.7–1.0)	0.68 ± 0.08 (0.5–0.9)	0.73 ± 0.11 (0.6–0.9)	< 0.001	< 0.001	< 0.001	0.049
Descending aortic diameter/MaxD	0.74 ± 0.08 (0.6–1.0)	0.62 ± 0.11 (0.4–1.0)	0.63 ± 0.10 (0.4–0.8)	< 0.001	< 0.001	0.0023	0.953
Diaphragm/MaxD	0.69 ± 0.10 (0.5–1.2)	0.56 ± 0.09 (0.4–0.9)	0.58 ± 0.10 (0.4–0.8)	< 0.001	< 0.001	0.0028	0.770
Ascending length/MaxD	2.06 ± 0.22 (1.7–2.7)	1.83 ± 0.21 (1.4–2.4)	2.80 ± 0.50 (2.1–3.8)	< 0.001	< 0.001	< 0.001	< 0.001

*MaxD* maximum thoracic ascending aorta diameter, *STJ* sinotubular junction

## Results

### Demographics

Between January 2019 and December 2024, 151 patients presented for ATAAD repair at our institution (Table S3). Of those, 13 (8.6%) had a predissection CTA within 3 years of dissection, comprising the predissection group. Patients with and without predissection CT did not differ significantly in age or sex. Patients with predissection CT were shorter (168.62 ± 8.32 vs. 174.70 ± 10.30 cm; *P* = 0.041).

The control group comprised 50 randomly selected patients without aortic disease or prior cardiac surgery (Table 1). The aneurysm group comprised 61 patients with MaxD ≥ 4.5 cm. Age and sex distribution differed overall among groups (*P* < 0.001 and *P* = 0.010, respectively). The aneurysm group was older than controls, but the age difference from the predissection group was not significant (*P* = 0.084). Male predominance was highest in the aneurysm group (aneurysm group, 70.5%; control group, 42.0%; predissection group, 61.5%; *P* = 0.010). Height was comparable across groups (*P* = 0.054).

### Aortic morphology

**Fig. 2:**
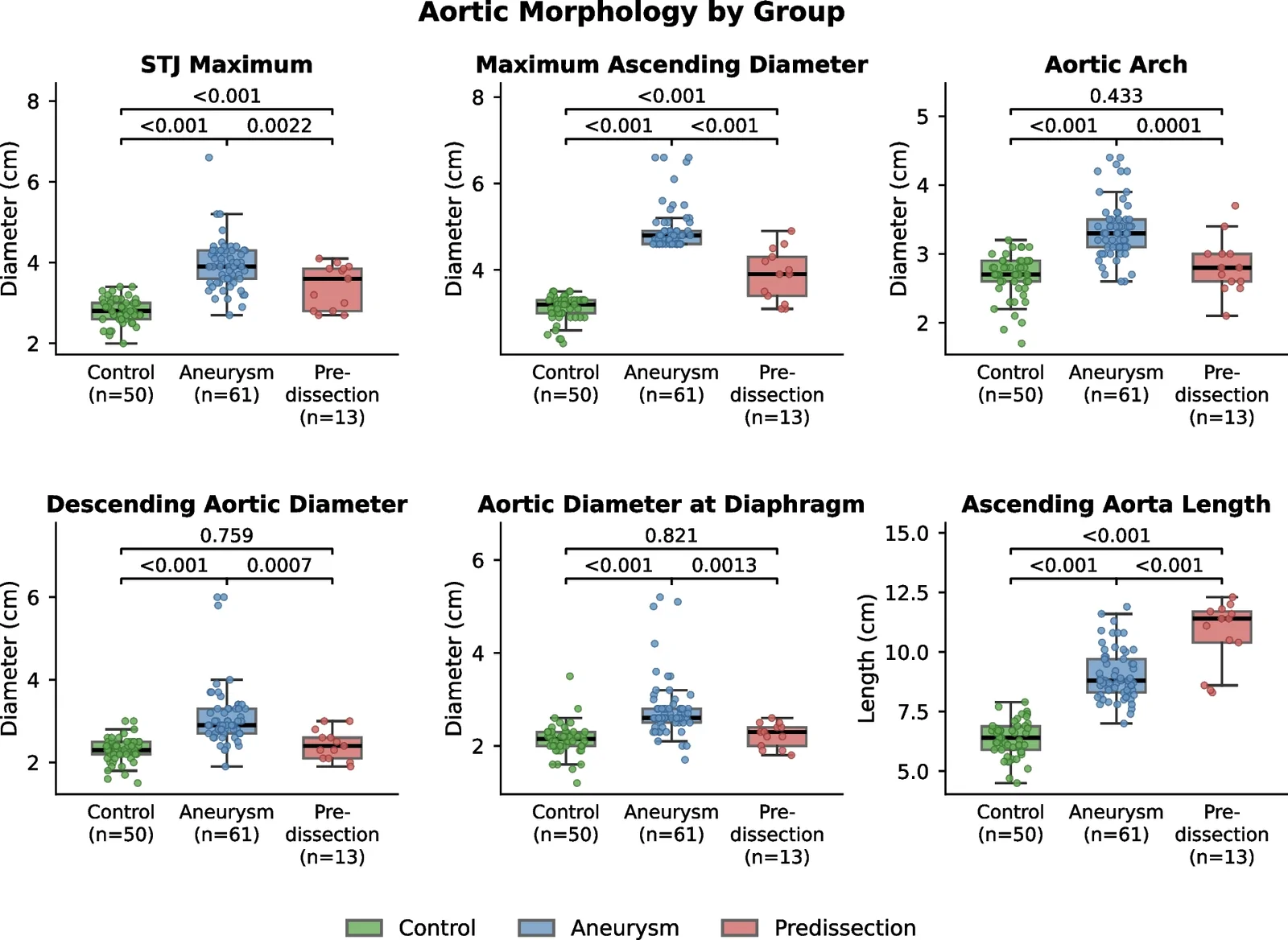
Aortic morphology by group. Box plots comparing raw aortic morphology measurements across the three study groups: control, aneurysm, and predissection. Each subplot displays the distribution of a single measurement. The sinotubular junction (STJ) maximum diameter represents the largest diameter at the STJ. The maximum ascending diameter (MaxD) is the largest diameter measured in the ascending aorta from the STJ to the origin of the innominate artery, with the STJ included in that region. Arch, descending, and diaphragm measurements represent maximal diameters at those anatomical levels. Ascending aorta length reflects the longitudinal extent from the STJ to the innominate artery origin. Individual data points are overlaid on each box plot. The horizontal line within each box represents the median; box boundaries represent the interquartile range (25th–75th percentile); whiskers extend to the 1.5 × interquartile range. Green: control; Blue: aneurysm; Red: predissection

Aortic morphology measurements by group are shown in Table 2 and Fig. 2*.* Diameters were analyzed from STJ through descending aorta. STJ diameter was significantly larger in the aneurysm group versus both control and predissection groups (control group, 2.81 ± 0.31 cm; aneurysm group, 3.94 ± 0.61 cm; predissection group, 3.41 ± 0.55 cm; both *P* < 0.001 vs. control; aneurysm group vs. predissection group, *P* = 0.0022). MaxD differed significantly (control group, 3.11 ± 0.30 cm; aneurysm group, 4.96 ± 0.52 cm; predissection group, 3.89 ± 0.60 cm; all pairwise *P* < 0.001). All predissection MaxD values were below 5.5 cm (maximum, 4.9 cm). Arch, descending, and diaphragm diameters were significantly larger in the aneurysm group compared to control and predissection groups (Table 2). Aortic dilation was diffuse throughout the thoracic aorta in the aneurysm group but localized predominantly to the ascending aorta in the predissection group. AAL was significantly longer in both aneurysm and predissection groups relative to control, and was significantly longer in the predissection group than in the aneurysm group as well. Control patients had a mean AAL of 6.38 ± 0.75 cm, compared to 9.03 ± 1.08 cm in the aneurysm group and 10.73 ± 1.41 cm in the predissection group (both *P* < 0.001 vs. control; aneurysm group vs. predissection group, *P* < 0.001).

### Aortic comparative morphology normalized to MaxD

**Fig. 3:**
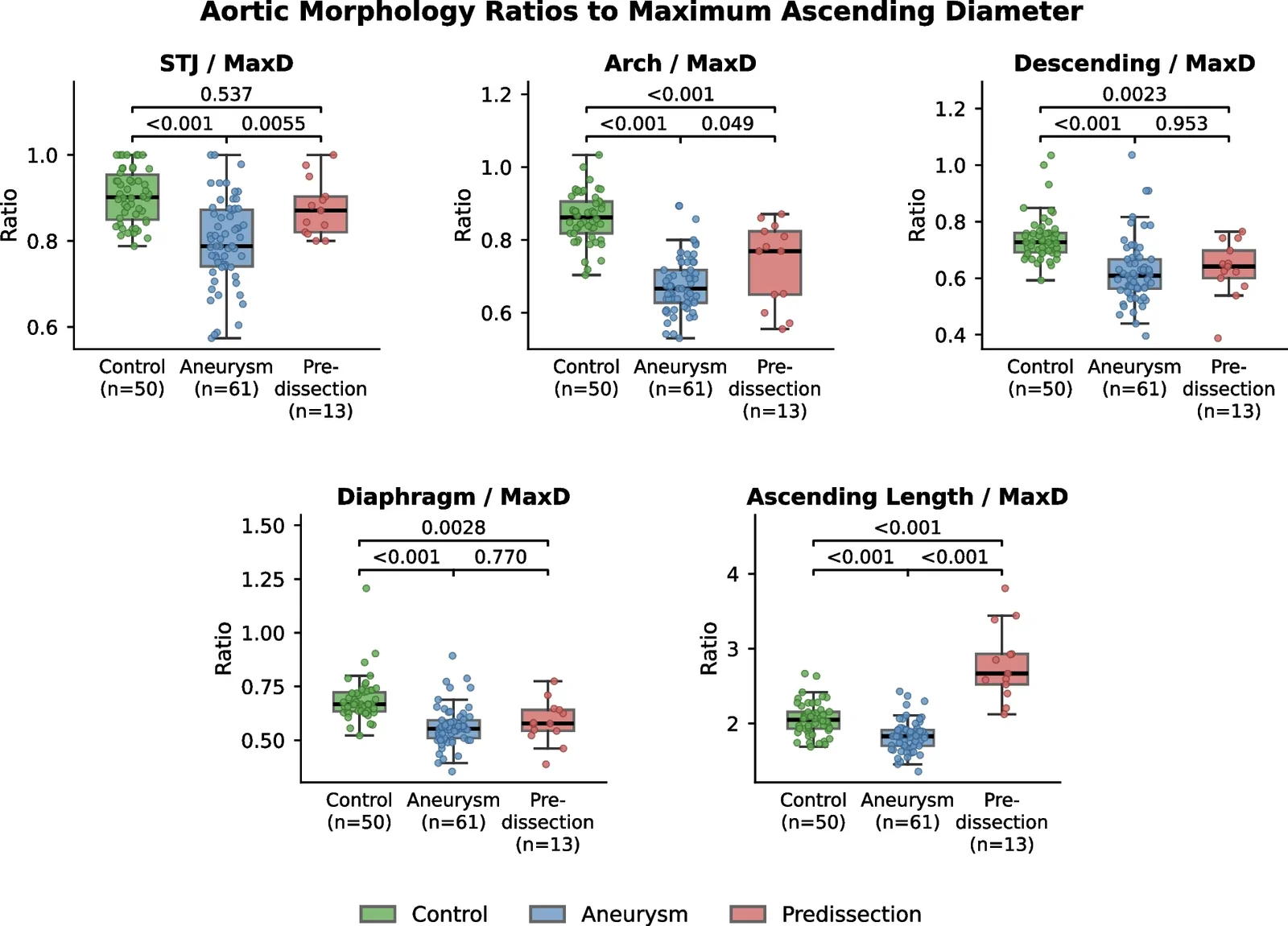
Aortic morphology ratios to maximum ascending diameter. Box plots displaying aortic segment diameters and ascending aorta length expressed as a ratio to maximum ascending diameter within each patient. The ascending length to maximum ascending diameter ratio is particularly relevant to the study hypothesis that the predissection group is characterized by a relatively elongated ascending aorta. Individual data points are overlaid on each box plot. The horizontal line within each box represents the median; box boundaries represent the interquartile range (25th–75th percentile); whiskers extend to the 1.5 × interquartile range. Green: control; Blue: aneurysm; Red: predissection

All measurements were normalized to MaxD to separate segment-specific differences from overall aortic size (Table 3, Fig. 3). Cross-diameter comparisons revealed group differences: STJ/MaxD (control group, 0.90 ± 0.06; aneurysm group, 0.80 ± 0.10; predissection group, 0.88 ± 0.07), Arch/MaxD (control group, 0.86 ± 0.07; aneurysm group, 0.68 ± 0.08; predissection group, 0.73 ± 0.11), descending/MaxD (control group, 0.74 ± 0.08; aneurysm group, 0.62 ± 0.11; predissection group, 0.63 ± 0.10), and diaphragm/MaxD (control group, 0.69 ± 0.10; aneurysm group, 0.56 ± 0.09; predissection group, 0.58 ± 0.10). The AAL/MaxD ratio differed between groups (control group, 2.06 ± 0.22; aneurysm group, 1.83 ± 0.21; predissection group, 2.80 ± 0.50), with differences between every pairwise comparison (all *P* < 0.001). To further characterize the discriminative performance of the AAL/MaxD ratio, receiver operating characteristic curve analysis was performed (Table S4). The area under the curve (AUC) for predissection versus control was 0.94 (95% confidence interval [CI], 0.87–0.99), with an optimal cutoff of 2.40 yielding sensitivity of 84.6% and specificity of 94.0%. The AUC for predissection versus aneurysm was 0.99 (95% CI, 0.97–1.00), with an optimal cutoff of 2.12 yielding sensitivity of 100% and specificity of 93.4%. For aneurysm versus control, the AUC was 0.79 (95% CI, 0.70–0.87) at a cutoff of 1.92. Given the small predissection cohort (*n* = 13), these results are exploratory.

### Dissection behavior

**Fig. 4:**
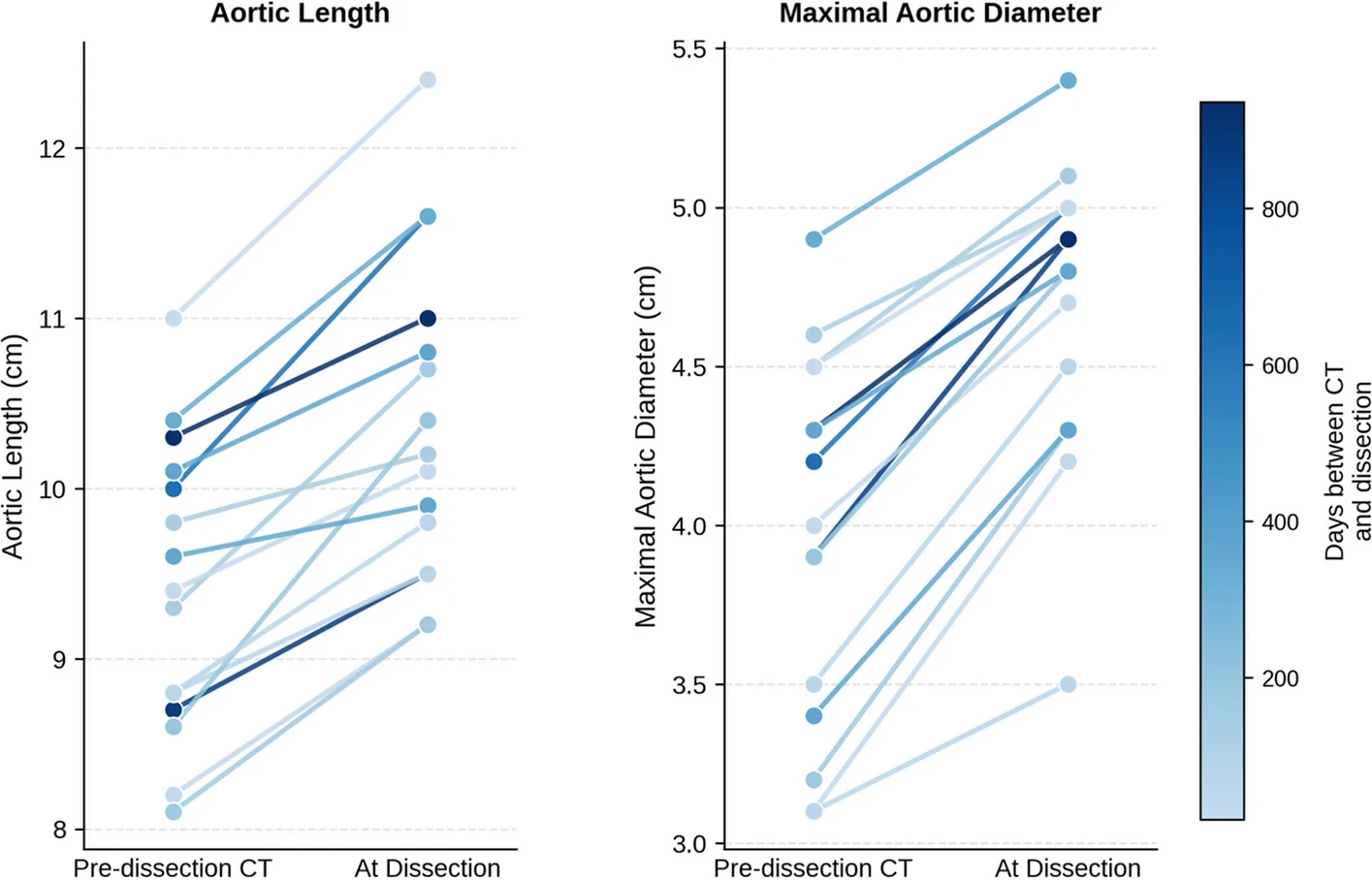
Changes in ascending aortic length and diameter between predissection and at dissection CT. Changes in ascending aortic morphology at the time of acute type A aortic dissection in the predissection subgroup (*n* = 13 with paired computed tomography [CT] imaging). **A** Ascending aorta length. **B** Maximum ascending aortic diameter. All patients demonstrated increases in both length and diameter. Ascending aortic length increased by a mean of 2.42 ± 0.84 cm (23.3% ± 9.3%; range, 1.3–4.1 cm). Maximum ascending aortic diameter increased by a mean of 0.85 ± 0.29 cm (22.9% ± 9.8%; range, 0.3–1.2 cm). All were statistically significant (both *P* < 0.001, paired t-test)

We examined our predissection group for change between pre-dissection and at-dissection CT (Fig. 4) and for the location of intimal disruption. The average increase in length and diameter was 23.3% and 22.9%, respectively. To determine whether the time interval between the predissection CT and the acute event influenced the magnitude of morphological change, we examined the correlation between the interscan interval and the change in both AAL and maximal diameter. No significant correlation with the interscan interval was identified for either measure (length: Pearson *r* = − 0.04, *P*** = **0.90; diameter: Pearson *r* = − 0.01, *P* = 0.98). The absence of a significant interval–change correlation should not be interpreted as excluding an effect of time; interval growth and changes occurring during dissection cannot be separated with these scans. The mean increase in aortic length was 2.42 ± 0.84 cm and in maximal diameter was 0.85 ± 0.29 cm, and these changes were observed consistently across patients with intervals ranging from 29 to 868 days. In no case was there a decrease in either measurement between the predissection and at-dissection CT scans. The intimal tear was identified radiographically in 11 of 13 patients. Of these 11 patients, nine were in the proximal TAA, one in the mid-TAA, and one at the base of the innominate artery.

## Discussion

We identified distinct radiographic and basic demographic patterns of thoracic aortic morphology among patients with normal aortas (control group), TAA aneurysms (aneurysm group), and patients with predissection imaging (predissection group). Key observations were as follows. Age and sex distribution differed overall among groups, whereas height did not differ significantly. The aneurysm group was significantly older than controls, but its age difference from the predissection group was not significant (Table 1). Across all groups, the aortic diameter decreased from the ascending aorta to the diaphragm (Table 2, Fig. 2). Compared with the control group, the aneurysm group had greater dilation throughout (STJ, ascending, arch, descending, and diaphragm). These differences were less pronounced in the predissection group. Both the predissection and aneurysm groups had an elongated aorta relative to the control group. Normalization of measurements to MaxD revealed that the AAL/MaxD ratio was markedly elevated in the predissection group, distinguishing it from the control and aneurysm groups (Table 3, S4). The ANCOVA model (group, age, sex, and height; *n* = 123 after excluding one aneurysm patient with missing height) confirmed group was the dominant predictor for both AAL and AAL/MaxD. For AAL, the predissection group remained significantly longer than the aneurysm group (β = + 1.92 cm, 95% CI 1.31–2.54, *P* < 0.001) and the control group significantly shorter (β = − 2.36 cm, 95% CI − 2.76 to − 1.96, *P* < 0.001); age was a significant but small covariate (β = + 0.018 cm/year, *P* = 0.005), while sex and height were not (*P* = 0.23 and *P* = 0.75). For AAL/MaxD, group effects were similarly robust (predissection vs. aneurysm β = + 1.00, 95% CI 0.83–1.16, *P* < 0.001; control vs. aneurysm β = + 0.27, 95% CI 0.16–0.37, *P* < 0.001), with no covariate reaching significance (all *P* > 0.28). In the predissection group, ascending aortic dimensions increased between the predissection and at-dissection CT scans, consistent with previously published studies [6, 9], and dissections occurred primarily in the proximal aorta (nine of the 11 with an identified tear). These findings suggest that patients who develop ATAAD may possibly exhibit a distinct geometric phenotype characterized by disproportionate longitudinal elongation but lesser dilation versus aneurysmal patients who have diffuse dilation and elongation.

The predissection group was numerically younger than the aneurysm group (59.15 ± 15.52 vs. 68.80 ± 10.86 years), but the difference was not significant (*P* = 0.084). In the IRAD study of 2,952 patients with type A dissection, the mean age was 61.5 ± 14.6 years and 67.5% were men [12]. Conversely, in a Mayo Clinic population study of thoracic aortic aneurysm, the median age was 77 years and 55% of the patients were women [13]. The direction of the age difference is consistent with these much larger studies.

However, ATAAD frequently occurs in aortas smaller than the size criteria for intervention in current guidelines [4, 5]. In IRAD, the mean maximal diameter measured at dissection was 5.3 cm, and 59% of type A dissections were below 5.5 cm [7]. In patients with a CT before dissection, the median maximal diameter was 45 mm and 96% were below 55 mm [8], and when predissection dimensions were estimated by modeling from scans at dissection, the median diameter was 35 mm [9]. In response, increasing attention has been directed toward aortic length. Several recent studies have shown that increased AAL is associated with adverse events [8, 9, 14–16]. Our study adds to this recent literature. In our cohort, all predissection MaxD values were below 5.5 cm (maximum, 4.9 cm). Patients did demonstrate an increase in AAL. This observation supports that longitudinal aortic remodeling may represent an important component of dissection pathophysiology.

The predissection and aneurysm cohorts exhibited elongated TAA relative to controls. AAL was greater in the predissection group than in the aneurysm group (10.73 ± 1.41 vs. 9.03 ± 1.08 cm; *P* < 0.001), but the ranges overlapped (8.3–12.3 vs. 7.0–11.9 cm). Disproportionate elongation may represent a form of longitudinal remodeling that increases axial wall stress without producing the degree of radial dilation typically used to define aneurysmal disease. Whether this longitudinal remodeling reflects a primary aortopathy or secondary adaptation to aging or systemic vascular risk factors cannot be determined from the present dataset, as clinical records were outside the scope of the ethical approval; this remains an important question for future prospective investigation. In our cohort, AAL increased by a mean of 2.42 cm (23.3%) between the predissection scan and the scan at dissection, which is larger than the 2.7% increase reported by Wu et al. [6]. Because this interval includes growth before dissection as well as change caused by the dissection, these scans cannot show how much of the elongation occurred before the event. We recognize that length is a more difficult parameter to measure, but advances in image processing have made it more readily available than previously. The use of centerline reconstruction has made length measurements more consistent (Fig. 1). In our study, because of the 3D shape of the annulus, defining the exact beginning of the annulus was less consistent than identifying the STJ. We therefore chose to measure from the STJ to the base/origin of the innominate artery.

Our findings suggest that aortic length is relevant and that multiaxial stress, including axial stress, should be considered alongside circumferential Laplace stress. These findings suggest that the morphological changes preceding dissection may reflect an inherent biomechanical vulnerability, consistent with prior observations of increased wall stiffness in predissection aortas [17, 18]. The aorta is a complex 3D structure exposed to multiaxial mechanical stresses. Computational modeling and finite element analyses have demonstrated that peak wall stress does not always occur at the location of maximal diameter, but rather at the curvature within the proximal TAA [19]. In one finite element analysis, the method predicted areas of high stress in dissected aortas co-located with intimal tears [19, 20]. Our findings are consistent with observations that most dissections occur in the proximal TAA [21].

Taken together, these findings are consistent with the hypothesis that ATAAD may be associated with more than one morphometric pattern: the classic aneurysmal phenotype characterized by diffuse dilation and elongation and a second phenotype characterized by disproportionate ascending elongation with only moderate dilation [4, 5, 19, 20]. Additionally, we propose an alternative framework for describing the aortas of patients who later dissected, based on distinct morphometric phenotypes beyond both diameter and length. Our findings suggest that a second phenotype, seen before dissection, is characterized by moderate dilation, disproportionate longitudinal elongation of the TAA, and relatively preserved geometry of the aortic arch and descending thoracic aorta. Importantly, the patients who later dissected may not represent a simple intermediate stage between normal and aneurysmal aortas.

### Limitations

This is a single-institution retrospective study with the inherent limitations of such a study design. The small sample size limits the generalizability of this work, which should be viewed as exploratory and hypothesis-generating and should be corroborated in the future with a larger cohort. The predissection group is the smallest of the three cohorts (*n* = 13), reflecting both the rarity of ATAAD and the strict inclusion criterion requiring a suitable CT in the prior 3 years; the resulting statistical power is limited. The predissection group is subject to an inherent selection bias, as it was restricted to patients for whom prior CT imaging could be identified and retrieved. While all CT studies in our facility were performed with ECG gating, gating status could not be confirmed for studies performed at outside facilities. Additionally, given the limitations of the ethical approval for this retrospective study, clinical comorbidity data such as hypertension and smoking history were not accessible. Several potentially relevant morphological features including aortic tortuosity, arch angulation, and total aortic volume were not included in the primary analysis. Future studies incorporating these indices may provide a more complete characterization of the predissection phenotype. The AAL/MaxD cutoffs and diagnostic performance estimates were derived in this small study cohort and have not been independently or prospectively validated. These findings should be regarded as hypothesis-generating, and larger multicenter studies will be needed. Further, all predissection measurements reflect a single static preoperative time point; morphological change at the time of dissection itself was assessed only in the subset with paired predissection and at-dissection imaging (Fig. 4), and longer-term post-dissection remodeling beyond that single follow-up point was not captured. Interobserver reproducibility was formally assessed in a random subset of patients (*n* = 72 accession-matched pairs, each measured twice) and was excellent for all measurements (ICC, 0.97–1.00) (Table S1), including the primary AAL/MaxD ratio (ICC, 0.99; 95% CI, 0.98–0.99).

## Conclusions

Our findings suggest that ascending aortic dissection may be associated with more than one distinct morphometric pattern; male sex was more frequent in both disease groups than in controls, although the difference was significant only for the aneurysm group. The commonly described aneurysmal phenotype is characterized by a slightly older population, dilation of the entire thoracic aorta, and aortic elongation. A second phenotype may be characterized by a slightly younger population (the age difference from the aneurysm group was not significant, *P* = 0.084), disproportionate longitudinal elongation of the ascending aorta, mild dilation of the ascending aorta, and relative preservation of arch and descending thoracic diameter. These observations are hypothesis-generating and require validation in larger, multicenter cohorts before informing clinical risk assessment. Incorporating multiple CT-based measures (morphology) to describe the ascending aorta into routine imaging reports and future risk models may warrant evaluation as a way to improve identification of patients at risk for acute dissection before catastrophic presentation.

## Supplementary Information

Additional file 1: Table S1. Interobserver measurement reproducibility. Table S2. Aortic morphology normalized to patient height (meters). Table S3. Comparison of patients with acute type A aortic dissection with and without predissection scans. Table S4. Diagnostic accuracy of the AAL/MaxD ratio for predissection phenotype identification.

## Data Availability

No datasets were generated or analysed during the current study.

## Abbreviations

AAL: Ascending aortic length
ANCOVA: Analysis of covariance
ANOVA: Analysis of variance
ATAAD: Acute type A aortic dissection
AUC: Area under the curve
CI: Confidence interval
CT: Computed tomography
CTA: Computed tomography angiography
ICC: Intraclass correlation coefficient
IRAD: International Registry of Acute Aortic Dissection
MaxD: Maximum thoracic ascending aorta diameter
MPR: Multiplanar reconstructions
STJ: Sinotubular junction
TAA: Thoracic ascending aorta
