# Supplementary material for: Radiographic phenotyping of healthy and diseased aortas
Source: J Cardiovasc Imaging. 2026 Oct 13;34:39. doi: 10.1186/s44348-026-00086-5 (PMC13660285; doi:10.1186/s44348-026-00086-5)
Supplement: Supplementary file 1 [file 44348_2026_86_MOESM1_ESM.docx]

**Supplemental Table 1.** **Inter-observer Measurement Reproducibility**

| **Variable** | **ICC(A,1)** | **95% CI** | **Mean Bias (cm)** | **Limits of Agreement (cm)** | **CV%** |
| --- | --- | --- | --- | --- | --- |
| **Diameter measurements** | | | | | |
| Sinotubular junction | 0.992 | [0.99, 0.99] | +0.02 | [-0.18, +0.22] | 2.0% |
| Ascending aorta diameter | 0.997 | [0.99, 1.00] | +0.03 | [-0.11, +0.17] | 1.1% |
| Aortic arch diameter | 0.974 | [0.96, 0.98] | +0.04 | [-0.16, +0.23] | 2.3% |
| Descending aorta diameter | 0.995 | [0.99, 1.00] | +0.03 | [-0.11, +0.16] | 1.7% |
| Aorta at diaphragm | 0.995 | [0.99, 1.00] | +0.03 | [-0.09, +0.14] | 1.6% |
| **Length measurement** | | | | | |
| Ascending aortic length | 0.987 | [0.98, 0.99] | +0.10 | [-0.41, +0.60] | 2.2% |
| **Derived ratio** | | | | | |
| AAL/MaxD ratio | 0.976 | [0.96, 0.98] | +0.01 | [-0.13, +0.15] | 2.5% |

Patients were remeasured by the independent observer (blinded to original values). ICC(A,1) = intraclass correlation coefficient, two-way absolute agreement, single measures. Mean bias = mean of (measurement 2 − measurement 1). LoA = limits of agreement (mean bias ± 1.96×SD of differences). CV% = coefficient of variation [SD of differences / (√2 × grand mean) × 100], allowing comparison across measurements of different scale. ICC ≥0.90 indicates excellent reproducibility.

# **Supplemental Table 2. Aortic Morphology Normalized to Patient Height (meters)**

| **Variable** | | **Control (n=50)** | **Aneurysm (n=61)** | **Predissection (n=13)** | **p (Overall)** | **Control vs Aneurysm** | **Control vs Predissection** | **Aneurysm vs Predissection** |
| --- | --- | --- | --- | --- | --- | --- | --- | --- |
|  | **STJ Maximum / Height (cm/m)** | 1.63 ± 0.18 | 2.26 ± 0.35 | 1.99 ± 0.32 | **<0.001** | **<0.001** | **<0.001** | **0.003** |
|  | **Maximum Ascending Diameter / Height (cm/m)** | 1.81 ± 0.18 | 2.84 ± 0.35 | 2.28 ± 0.41 | **<0.001** | **<0.001** | **<0.001** | **<0.001** |
|  | **Aortic Arch / Height (cm/m)** | 1.56 ± 0.18 | 1.93 ± 0.29 | 1.71 ± 0.25 | **<0.001** | **<0.001** | 0.084 | **0.006** |
|  | **Descending Aortic Diameter / Height (cm/m)** | 1.34 ± 0.17 | 1.79 ± 0.50 | 1.47 ± 0.26 | **<0.001** | **<0.001** | 0.463 | **0.007** |
|  | **Aortic Diameter at Diaphragm / Height (cm/m)** | 1.25 ± 0.20 | 1.60 ± 0.42 | 1.37 ± 0.30 | **<0.001** | **<0.001** | 0.406 | **0.033** |
|  | **Ascending Aorta Length / Height (cm/m)** | 3.72 ± 0.45 | 5.18 ± 0.68 | 5.45 ± 0.57 | **<0.001** | **<0.001** | **<0.001** | 0.238 |

Values presented as mean ± SD. P-values from one-way ANOVA with Tukey's HSD post-hoc pairwise comparisons. Significant p-values (p<0.05) shown in red bold.

**Supplemental Table 3. Comparison of Patients with and without dissections stratified by predissection scans.**

|  | **Dissection w/o scan n = 140** | **Pre Dissection w/ scan n = 13** | **P-Value** |
| --- | --- | --- | --- |
| **Age** | 61.26 ± 15 (22–88) | 58.19 ± 17.61 (22–79) | 0.44 |
| **Height** | 174.7 ± 10.3 (149.9–205.7) | 170.56 ± 8.64 (157–183) | 0.12 |
| **Male Gender** | 73.19% | 62.5% | 0.06 |

**Supplemental Table 4: Diagnostic Accuracy of the AAL/MaxD ratio for Predissection Phenotype Identification.**

| **Comparison** | **AUC (95% CI)** | **Optimal Cutoff (AAL/MaxD)** | **Sensitivity** | **Specificity** |
| --- | --- | --- | --- | --- |
| **Predissection vs Control (n=13 vs n=50)** | 0.943 (0.870–0.994) | 2.40 | 84.6% | 94.1% |
| **Predissection vs Aneurysm (n=13 vs n=61)** | 0.986 (0.961–1.000) | 2.12 | 100.0% | 92.3% |
| **Aneurysm vs Control (n=61vs n=50)** | 0.788 (0.704–0.865) | 1.92† | 76.9% | 76.5% |

AAL/MaxD = Ascending Aortic Length divided by Maximum Ascending Aortic Diameter. AUC=Area under the Curve. Optimal cutoff values determined by the Youden index (maximizing sensitivity + specificity − 1). 95% confidence intervals derived by 2,000-iteration bootstrap resampling. †For Aneurysm vs Control, AAL/MaxD < 1.92 identifies the Aneurysm phenotype (lower ratio reflects diffuse dilation relative to length).
